# Effect of Supplementation of Tanshinone IIA and Sodium Tanshinone IIA Sulfonate on the Anticancer Effect of Epirubicin: An *In Vitro* Study

**DOI:** 10.1155/2011/841564

**Published:** 2011-05-24

**Authors:** Szu-Erh Chan, Hung-Wen Lai, Chin-Cheng Su, Shou-Jen Kuo, Su-Yu Chien, Hui-Yi Lin, Dar-Ren Chen

**Affiliations:** ^1^Department of Surgical Medicine, Erlin Branch of Changhua Christian Hospital, Changhua 526, Taiwan; ^2^Comprehensive Breast Cancer Center, Changhua Christian Hospital, Changhua 500, Taiwan; ^3^Department and Institute of Pharmacology, School of Medicine, National Yang-Ming University, Taipei 112, Taiwan; ^4^School of Medicine, College of Health Care and Management, Chung Shan Medical University, Taichung 40201, Taiwan; ^5^School of Nutrition, College of Health Care and Management, Chung Shan Medical University, Taichung 40201, Taiwan; ^6^Department of Pharmacology, Changhua Christian Hospital, Changhua 500, Taiwan; ^7^Department of Pharmacology, School of Pharmacology, China Medical University, Taichung 40402, Taiwan

## Abstract

Tanshinone IIA (Tan IIA) and sodium tanshinone IIA sulfonate (STS) were found to have protective effects on cardiomyocyte against adriamycin-induced damage and may be used clinically. It is unclear whether the supplementation of STS or Tan IIA would affect the anticancer activity of anthracycline. To evaluate the effect of Tan IIA or STS on the anticancer of epirubicin, the cell viability, apoptosis, Akt expression, and uptake of epirubicin after supplementation of Tan IIA or STS in the epirubicin-treated BT-20 cells were measured and compared. Tan IIA inhibited BT-20 cell growth and induced apoptosis in a time- and dose-dependent manner. When Tan IIA was used with epirubicin, an increase of BT-20 cells apoptosis was accompanied by the decreasing phosphorylation of Akt. STS had no effect on the cell viability of BT-20 cells. However, when used with epirubicin, STS decreased the epirubicin-induced cytotoxicity and apoptosis in BT-20 cells. The antagonistic effect of STS on epirubicin-induced cytotoxicity in BT-20 cells occurred concomitantly with the reduced epirubicin uptake and the increased phosphorylation of Akt. STS decreased the uptake of epirubicin in BT-20 cells and blocked epirubicin-induced apoptosis through activation of Akt.

## 1. Introduction

Breast cancer is one of the leading causes of cancer death among women all over the world; it accounts for approximately 30% of all new cancer cases each year, with an annual incidence of approximately 200,000 in the United States [1]. In Taiwan, the incidence of breast cancer has increased steadily from 10/10^5^ in 1979 to 50/10^5^ in 2006, and it is now the most commonly occurring cancer and leading cause of death among Taiwanese women [2]. Anthracyclines such as doxorubicin and epirubicin are highly efficacious against breast cancer [3], and are commonly used in adjuvant or neoadjuvant chemotherapy to reduce local recurrence or distant metastasis [4, 5]. However, their widespread use was limited by their cardiotoxicity, which was irreversible and cumulatively dose related [6–12].

Danshen, a traditional Chinese medicine derived from the dry root or rhizome of *Salviae miltiorrhizae Bge*, is widely used in China and other countries, including the United States [13, 14]. The reported cardioprotective effects of Danshen include improving microcirculation [15], suppressing the formation of thromboxane [16], inhibiting platelet adhesion and aggregation [17], and protecting against myocardial ischemia [13], as well as its angiotensin II-induced hypertrophic response [18, 19]. Among the more than 30 diterpene compounds, tanshinone IIA (Tan IIA), a lipid-soluble compound, and sodium tanshinone IIA sulfonate (STS), the water-soluble derivative of tanshinone IIA, are the two most well-known and abundant components of Danshen [13, 20, 21].

The exact mechanisms of anthracycline-related cardiotoxicity are not fully understood, but it has been suggested that oxidative stress, oxygen-free radicals, iron chelation, and lipid peroxidation may play important roles [7, 12, 22]. Tan IIA and STS were reported to have antioxidant properties, which could break the chain reactions of peroxidation by scavenging lipid-free radicals [20], and increase the activity of superoxide dismutase (SOD) [23]. Recent studies have revealed that Tan IIA [24, 25] and STS [26, 27] have the protective effects of cardiomyocyte against adriamycin-induced lipid peroxidation and oxidative stress-mediated apoptosis in preclinical experiments. The cardioprotective effect of Tan IIA and STS against adriamycin-related cardiotoxicity might be a potential therapeutic agent for clinical use.

For a cardioprotective agent to be used clinically, it is important to confirm that it will not impair the anticancer effect of chemotherapy [28]. At present, it is unclear whether the supplementation of STS or Tan IIA would affect the anticancer effect of anthracyclines, such as epirubicin. Therefore, the objective of this *in vitro* study was to elucidate the influence of STS or Tan IIA on the epirubicin-induced cytotoxicity of breast cancer cells.

## 2. Materials and Methods

### 2.1. Cell Lines and Culture

The human breast cancer cell line BT-20 was obtained from the American Type Culture Collection (ATCC). BT-20 breast cancer cells were maintained at 37°C in 5% CO_2_ and 95% air and grown in RPMI 1640 medium supplemented with 10% fetal bovine serum, 100 IU/mL penicillin, and 100 mg/mL streptomycin (Invitrogen, Carlsbad, CA).

### 2.2. Compounds

STS and Tan IIA purchased from Herbasin Co., Ltd. (Shenyang, China) were dissolved in PBS and DMSO separately, at 2 mg/mL and 5 mg/mL, and used as stock. Epirubicin 150 mg/vial was purchased from Pharmacia & Upjohn S.P.A. (Milan, Italy) and dissolved in PBS at 30 mg/mL as stock.

### 2.3. Cell Viability Assay

Cell viability was evaluated by the WST-1 assay (BioVision, Mountain View, CA). The WST-1 assay measures the activity of mitochondrial dehydrogenases of viable cells by reducing tetrazolium salt to formazan. Briefly, cells (1 × 10^4^/well) were seeded in 96-well plates overnight, followed by epirubicin and/or STS (or Tan IIA) treatment for 72 hours. Then, WST-1 was added to each well, and the cells were incubated at 37°C for 4 hours. Finally, plates were measured at OD450 and referenced at OD620 with a microplate reader (SunriseTM, Tecan Groug LTD, Austria). At least three wells per group were analyzed, and the procedure was repeated three times.

### 2.4. Apoptosis Analyses

BT-20 cells were plated in 12-well plates (6 × 10^5^/well) and incubated with STS or Tan IIA at different concentrations for 48–72 hours. The cells were then harvested by centrifugation, fixed with 70% ethanol (in PBS) at 4°C overnight, and resuspended in PBS containing 4 *μ*g/mL of propidium iodide (PI) (Sigma), 0.1 mg/mL RNase A, and 0.1% Triton X-100 in the dark. The cell suspension was incubated at 37°C for 30 minutes. The cell cycle was determined and analyzed with flow cytometry (Beckman Coulter, USA) equipped with an argon ion laser at a 488 nm wavelength.

### 2.5. Determination of Epirubicin Uptake

Epirubicin 1 *μ*g/mL was used for the measurement of uptake of BT-20 cells by flow cytometer (Becton Dickinson FACScan), using laser emitting at 488 nm, close to the absorption maximum of epirubicin [29]. BT-20 cells were plated in 12-well plates (9∗10^4^/well) and incubated with epirubicin 1 *μ*g/mL and/or STS (or Tan IIA) treated with different concentrations for 24 hours. The cells were then harvested by centrifugation and fixed with 4% paraformaidehyde at 4°C overnight, in the dark. The fluorescence intensity increased paralleled with the uptake of epirubicin into BT-20 cells and was used to determine the cell uptake of epirubicin.

### 2.6. Determination of Combinatorial Effects

The ability of STS (or Tan IIA) and epirubicin to act in a synergistic, additive, or antagonistic manner with regard to growth inhibition was determined by a combination index (CI) as proposed by Chou and Talalay [30, 31]. Calcusyn software (Biosoft, Great Shelford, Cambridge, UK) was used to determine the CI for each concentration of drug mixture used. A value of CI < 1 represents a case in which a synergism of epirubicin and STS (or Tan IIA) was present. CI values of 1 and >1 represent additive and antagonistic effects, respectively.

### 2.7. Akt Activation in the Presence or Absence of Tan IIA or STS in Epirubicin-Treated BT-20 Breast Cancer Cells

Constitutive Akt signaling appears to play an important role in the proliferative activity, resistance to chemotherapy, and hormone therapy of breast cancer cells [32–34]. We further surveyed whether an Akt-related pathway was involved in the antiapoptotic effect conferred by STS (or Tan IIA) in epirubicin-treated breast cancer cells. After drug treatment for 24 hours, cells were harvested and lysed with the lysis buffer (20 mM Tris pH 7.5, 150 mM NaCl, 1 mM EDTA, 1 mM EGTA, 1% Triton X-100, 2.5 mM sodium pyrophosphate, 1 mM glycerolphosphate, 1 mM Na_3_VO_4_, 1 mM phenylmethylsulfonyl fluoride, 10 mg/mL leupeptin and 10 mg/mL aprotinin). The lysates were quantified by Bradford assay and heated at 95°C for 5 minutes in a sample buffer. Fifty micrograms of protein were separated by 8% polyacrylamide gels and transferred to PVDF membranes (Millipore, Bedford, MA). The membranes were blocked for one hour with 5% nonfat dried milk in 0.1% PBST (500 mL 1X PBS with 0.5 mL Tween-20) at room temperature, and then were incubated with Akt, phosphorylated Akt (phospho-Akt), JNK, phosphorylated JNK (phospho-JNK), p38, phosphorylated p38 (phospho-p38), MAPK, and phosphorylated MAPK (phospho-MAPK) specific primary antibodies overnight at 4°C. Later, membranes were washed three times with PBST and incubated with horseradish peroxidase- (HRP-) conjugated secondary antibody for one hour at room temperature. After washing with PBS three times, the protein signals were detected by an enhanced chemiluminescence (ECL) detection system (Amersham Biosciences, Piscataway, NJ). The band densities were quantified by using Image J software (http://rsb.info.nih.gov/ij/) and normalized the ratio of phospho-AKT/total-AKT, phospho-JNK/total-JNK, phospho-p38/total p38, and phospho-MAPK/MAPK to *β*-actin for protein loading.

Antibody for Akt, phospho-Akt, JNK, phospho-JNK, p38, phospho-p38, MAPK, and phospho-MAPK were purchased from Cell Signaling TECHNOLOGY. HRP-conjugated goat antimouse and mouse antirabbit IgG antibodies were obtained from Amersham (Freiburg, Germany). Rhodamine-conjugated rabbit antimouse was obtained from Jackson Laboratory (West Grove, WI). Anti-*β*-actin antibody was obtained from Sigma (St. Louis, MO). Other materials and reagents not specified were obtained from Sigma or Merck.

### 2.8. Statistical Analyses

Analyses were performed using the Statistics Analysis System (SAS 9.1). Data are presented as mean ± standard deviation except where indicated. Comparisons between groups were analyzed using the chi-square test, Student's two-tailed *t* test, or one-way analysis of variance (ANOVA) with Bonferroni's correction as appropriate. A value of *P* < .05 was considered statistically significant.

## 3. Results

### 3.1. Effect of STS or Tan IIA on Growth of BT-20 Breast Cancer Cells

**Figure 1:**
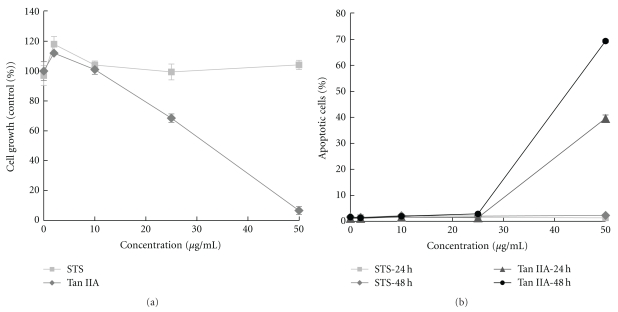
Effect of Tan IIA or STS on the growth of BT-20 breast cancer cells. (a) BT-20 cells were treated with 2, 10, 25, and 50 *μ*g/mL of Tan IIA or STS for 72 h. The cell growth was determined by WST-1 assay. (b) BT-20 cells were treated with 2, 10, 25, and 50 *μ*g/mL of Tan IIA or STS for 24 h or 48 h. The ratio of apoptosis was determined by flow cytometry. Data are the mean ± SD of triplicates from a representative assay of three separate experiments.

To evaluate the effect of STS or Tan IIA on breast cancer cells *in vitro*, BT-20 breast cancer cells were exposed to 2–50 *μ*g/mL of Tan IIA or STS for 72 hours. The cell viability was determined by WST-1 assay. As shown in Figure 1(a), after treatment with 2–50 *μ*g/mL of Tan IIA, the growth of BT-20 cells decreased in a dose-dependent manner. In contrast, STS had no significant effect on the growth of BT-20 breast cancer cells.

### 3.2. Apoptotic Effect of STS or Tan IIA on BT-20 Breast Cancer Cells

In the cell cycle analysis, treatment of BT-20 cells with 1 and 10 *μ*g/mL of Tan IIA did not result in any significant apoptosis, while treatment of BT-20 cells with 50 *μ*g/mL Tan IIA resulted in a 70% increase of apoptosis at 48 hours as compared with the control (Figure 1(b)). No apparent apoptosis was found with STS, even at concentrations of 50 *μ*g/mL.

### 3.3. Supplementation of STS (or Tan IIA) in Epirubicin-Treated BT-20 Breast Cancer Cells

**Figure 2:**
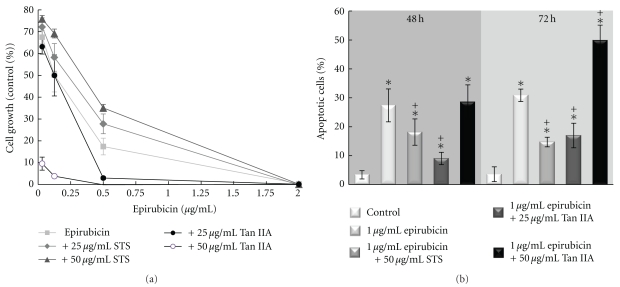
Effect of Tan IIA or STS on epirubicin-induced cytotoxicity and apoptosis in BT-20 breast cancer cells. (a) BT-20 breast cancer cells were treated with 0–2 *μ*g/mL epirubicin in the presence or absence of STS (0–50 *μ*g/mL) or Tan IIA (0–50 *μ*g/mL) for 72 h. The cell growth was determined by WST-1 assay. (b) BT-20 cells were treated with 1 *μ*g/mL of epirubicin. The change in apoptosis with the addition of STS (50 *μ*g/mL) and Tan IIA (2, 10, 25, 50 *μ*g/mL) in epirubicin- (Epi-) treated BT-20 cells was determined by flow cytometry at 48 or 72 h. *P < .05 when compared with the control group; ^+^ *P* < .05 when compared with the epirubicin group. Data are the mean ± SD of triplicates from a representative assay of three separate experiments.

The BT-20 breast cancer cell line used here was sensitive to epirubicin in a dose-dependent manner. When the concentration of epirubicin reached 2 *μ*g/mL, almost all the BT-20 breast cancer cells were nonviable (Figure 2(a)). Supplementation with STS increased the cell viability of BT-20 cells under epirubicin treatment, while Tan IIA potentiated the cytotoxicity of epirubicin on BT-20 cells.

The addition of 50 *μ*g/mL STS and 25 *μ*g/mL Tan IIA to 1 *μ*g/mL epirubicin-treated BT-20 cells decreased epirubicin-induced apoptosis (Figure 2(b)). The combination of 50 *μ*g/mL Tan IIA and 1 *μ*g/mL epirubicin resulted in higher apoptosis in BT-20 cells than epirubicin alone.

The combination effect of epirubicin with STS (or Tan IIA) on BT-20 cells was analyzed with Calcusyn. The combination of epirubicin with STS, most of the time, exerted an antagonistic effect. While combination of epirubicin with Tan IIA resulted in a more complex synergistic, additive or antagonistic effect at different drug concentration combinations (Table 1).

### 3.4. Uptake of Epirubicin in BT-20 Cells in the Presence or Absence of STS or Tan IIA

**Figure 3:**
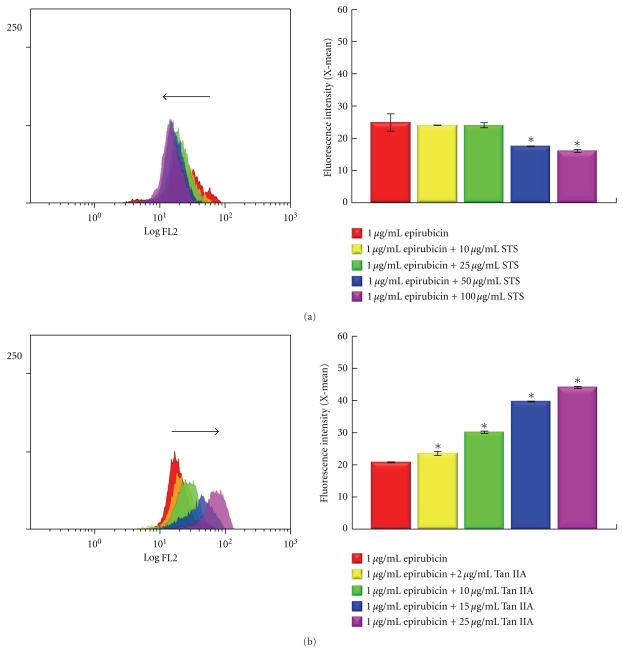
Uptake of epirubicin in BT-20 breast cancer cells in the presence or absence of STS (a) or Tan IIA (b) treatment for 24 h. Data are the mean ± SD of triplicates from a representative assay of three separate experiments. *P < .05 as compared with 1 *μ*g/mL epirubicin-treated BT-20 cells.

We then determined the uptake of epirubicin in BT-20 cells before and after supplementation of STS or Tan IIA, using flow cytometry. When cotreated with STS, the uptake of epirubicin in BT-20 breast cancer cells was decreased in a dose-dependent manner (Figure 3(a)). In contrast, Tan IIA dose-dependently increased the uptake of epirubicin in BT-20 breast cancer cells (Figure 3(b)).

### 3.5. Activation of the Akt-Related Pathway with the Supplementation of STS (or Tan IIA) in Epirubicin-Treated BT-20 Breast Cancer Cells

**Figure 4:**
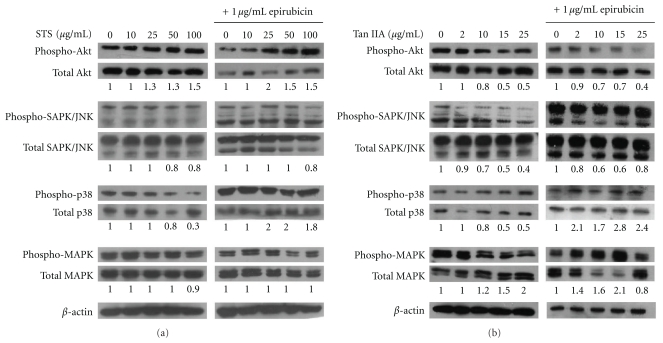
Akt-related pathway activation in the presence or absence of STS (or Tan IIA) in epirubicin-treated BT-20 breast cancer cells for 24 h. (a) Representative results of Western blotting showing changes in the levels of Akt, phospho-Akt, JNK, phospho-JNK, P38, phospho-p38, MAPK, and phospho-MAPK in BT-20 cells treated with STS and/or 1 *μ*g/mL epirubicin. (b) Representative results of Western blotting showing changes in the levels of Akt, phospho-Akt, JNK, phospho-JNK, P38, phospho-p38, MAPK, and phospho-MAPK in BT-20 cells treated with Tan IIA and/or 1 *μ*g/mL epirubicin.

The expression of Akt, phospho-Akt, JNK, phospho-JNK, p38, phospho-p38, MAPK, and phospho-MAPK with the supplementation of STS or Tan IIA to epirubicin-treated BT-20 breast cancer cells was evaluated by Western blot. When STS was added, the level of phospho-Akt was increased along with the increase of dose (Figure 4(a)). In contrast, Tan IIA treatment of BT-20 cells resulted in a decreased expression of phospho-Akt in a dose-dependent manner (Figure 4(b)). The activation of Akt in epirubicin-treated BT-20 breast cancer cells was found in the STS + epirubicin group, but not in the Tan IIA+epirubicin group. The phosphorylated MAPK, JNK, and p38 were slightly increased in the treatment of BT-20 cells with STS, or epirubicin combined with STS. On the contrary, the phosphorylation of MAPK, JNK, and p38 were slightly decreased in BT-20 cells when treated with Tan IIA, or the combination of Tan IIA with epirubicin.

## 4. Discussion

Our study was designed to test the effect of supplementation of STS or Tan IIA on the anticancer effect of epirubicin in an *in vitro* BT-20 cell line model. In our study, STS alone had no effect on growth inhibition or apoptosis in BT-20 breast cancer cells, while Tan IIA could dose-dependently reduce BT-20 breast cancer cell viability and induce apoptosis. The supplementation of STS to epirubicin-treated BT-20 cells reduced epirubicin-related cytotoxicity and apoptosis. The supplementation of Tan IIA to epirubicin-treated BT-20 breast cancer cells potentiated the cytotoxic effect of epirubicin. The antagonistic effect of STS on epirubicin-induced cytotoxicity in BT-20 breast cancer cells occurred concomitantly with the reduced uptake of epirubicin and increased phosphorylation of Akt.

Epirubicin, a commonly used chemotherapeutic agent in adjuvant or neoadjuvant settings to reduce local recurrence or distant metastasis [4, 5], is highly efficacious against breast cancer; a concentration of epirubicin as low as 0.2 *μ*g/mL could reduce BT-20 breast cancer cell growth by 50%. Tan IIA, the lipophilic extract of Danshen, had a growth-inhibiting effect on BT-20 breast cancer cells and induced apoptosis in a dose-dependent manner. The anticancer effect of Tan IIA has been reported in acute promyelocytic leukemia [35], human glioma cells [36], breast cancer cells [37], hepatoma cells [38], and vascular endothelial cells [39]. STS, the water soluble form of Tan IIA, did not have a growth inhibitory effect, nor did it induce apoptosis, even at a 50 *μ*g/mL concentration.

The influence of the supplementation of STS or Tan IIA on the anticancer effect of epirubicin was unclear. In our *in vitro* cell line study, the supplementation of STS to epirubicin-treated BT-20 cells reduced the cell growth inhibitory effect of epirubicin and interfered with epirubicin-induced apoptosis. The supplementation of Tan IIA to epirubicin-treated BT-20 breast cancer cells potentiated the cytotoxic effect of epirubicin (Figure 2(a)). Although a decrease of epirubicin-related apoptosis was found when Tan IIA (25 *μ*g/mL) was used with epirubicin (Figure 2(b)), the overall cell viability was decreased. This discrepancy might be related to cell death other than apoptosis, like an increase of necrosis, autophage [40], or cell cycle arrest.

The interaction of STS or Tan IIA with epirubicin was further evaluated by the Calcusyn software, which is commonly used for the analysis of drug combination effect. STS when used with epirubicin had an antagonistic effect, while interaction of Tan IIA with epirubicin is more complex. In most circumstances, STS when used with epirubicin would result in an antagonistic effect. When Tan IIA was used with epirubicin, most of the time there would be an additive effect. The synergistic effect was found when 0.25 *μ*g/mL epirubicin was used with 25 *μ*g/mL Tan IIA. However, an antagonistic effect was present when high concentration (≥1 *μ*g/mL) of epirubicin was used with Tan IIA. The antagonistic effect of STS on chemotherapy-related cytotoxicity in breast cancer cells is rarely reported. Since an antagonistic effect between drugs would cause a suboptimal therapeutic effect, it is important that combining epirubicin with STS should be avoided.

A drug's successful entrance into cells is the first step in the eradication of malignant cancer cells. Failure of a drug to pump into cells, or the ability of cancer cells to actively efflux drugs, leads to a decrease in cellular drug accumulation to a subtoxic level and is frequently related to the development of drug resistance [41, 42]. We used flow cytometry to determine the uptake of epirubicin in BT-20 cells before and after supplementation of STS or Tan IIA. When cotreated with STS, the uptake of epirubicin in BT-20 breast cancer cells was decreased in a dose-dependent manner. In contrast, Tan IIA dose-dependently increased the uptake of epirubicin in BT-20 breast cancer cells. These findings were compatible with the observation that the supplementation of STS will interfere with the epirubicin-related anticancer effect, while the combination of Tan IIA with epirubicin would potential the cytotoxic effects of epirubicin.

Constitutive Akt signaling plays an important role in cell proliferation and resistance to hormone or chemotherapy in breast cancer [32–34]. The STS dose-dependently increased the phosphorylation of Akt in BT-20 breast cancer cells. In the epirubicin-treated BT-20 cells, the supplementation of STS also dose-dependently increased the phosphorylation of Akt. The upregulation of Akt renders BT-20 cells more resistant to epirubicin-related cytotoxicity. This could partly explain the antagonistic effect of STS when combined with epirubicin. On the other hand, Akt phosphorylation in BT-20 breast cancer cells was decreased after supplementation with Tan IIA. This was consistent with the additive and synergistic cytotoxic effect of Tan IIA and epirubicin in BT-20 breast cancer cells.

STS may be an active cardioprotectant, as revealed in previous reports [19, 20, 23, 26, 27], although none of them discussed the interaction between STS and epirubicin. Our preclinical result showed that STS may interfere with the anticancer effect of epirubicin. The decrease in epirubicin-induced BT-20 breast cancer cell cytotoxicity after supplementation with STS may be due to the decreased uptake of epirubicin and the activation of Akt. One possibility is that STS had a similar effect on cardiomyocytes in blocking the uptake of epirubicin and activating the Akt pathway. If this is the case, then STS may not be an ideal cardioprotectant in patients undergoing chemotherapy with anthracycline. The cardioprotective [24, 25] and anticancer [35–39] effects of Tan IIA, without disturbing the anticancer effect of epirubicin, supported the possible role of Tan IIA as a cardioprotectant. However, our study was limited, in that we did not perform an *in vivo* study to further confirm the effects of STS and Tan IIA observed in the *in vitro* breast cancer cell line.

## Figures and Tables

**Table 1:** Interaction between STS (or Tan IIA) and epirubicin on the growth of BT-20 breast cancer cells.

Epirubicin (*μ*g/mL)	STS (*μ*g/mL)	Tan IIA (*μ*g/mL)	Fa	CI	Effect
0.03125	25		0.048	2.014	Antagonistic
0.0625	25		0.0313	3.931	Antagonistic
0.125	25		0.0686	3.314	Antagonistic
0.25	25		0.0869	4.713	Antagonistic
0.5	25		0.1447	5.626	Antagonistic
1	25		0.2323	6.95	Antagonistic
2	25		0.7902	2.038	Antagonistic

0.03125	50		0.0492	3.166	Antagonistic
0.0625	50		0.0351	5.15	Antagonistic
0.125	50		0.0776	3.825	Antagonistic
0.25	50		0.0683	6.652	Antagonistic
0.5	50		0.2	4.557	Antagonistic
1	50		0.423	3.733	Antagonistic
2	50		0.8946	1.131	Additive/antagonistic

0.03125		25	0.6109	1.066	Additive/antagonistic
0.0625		25	0.5097	1.302	Additive/antagonistic
0.125		25	0.6354	1.197	Additive/antagonistic
0.25		25	0.8371	0.902	Synergistic
0.5		25	0.8257	1.141	Additive/antagonistic
1		25	0.8341	1.511	Antagonistic
2		25	0.8926	1.708	Antagonistic

0.03125		50	0.9412	1.013	Additive/antagonistic
0.0625		50	0.9422	1.018	Additive/antagonistic
0.125		50	0.9392	1.057	Additive/antagonistic
0.25		50	0.9382	1.106	Additive/antagonistic
0.5		50	0.9366	1.205	Additive/antagonistic
1		50	0.9372	1.377	Antagonistic
2		50	0.9374	1.728	Antagonistic

Fa: fraction affected; CI: combination index.

A value of CI <1 represents a case where synergism of epirubicin and STS (or Tan IIA) was present. CI values of 1 and >1 represent additive and antagonistic effects, respectively.
